# Synthetic relationships with Social Pedagogical Agents in education: a scoping literature review

**DOI:** 10.3389/frai.2026.1625438

**Published:** 2026-03-02

**Authors:** Sebas Nouwen, Janienke Sturm, Uwe Matzat, Wijnand IJsselsteijn

**Affiliations:** 1 Department of Industrial Engineering & Innovation Sciences (IEIS), Eindhoven University of Technology, Eindhoven, Netherlands; 2 Human Technology Interaction (HTI) Research Group, Fontys University of Applied Sciences, Eindhoven, Netherlands

**Keywords:** conversational agents, education, Pedagogical Agents, relationship, social AI, students, synthetic relationship, virtual agents

## Abstract

**Introduction:**

As AI in education increasingly takes the form of Social Pedagogical Agents (SPAs), learners begin to relate to these systems in human-like ways. Concepts such as social presence, affective support, trust, and rapport are widely studied but scattered across domains, labels, and measures. This scoping review maps how “synthetic relationships” between students and SPAs are described in empirical educational research and how SPA design and interaction features relate to students’ learning experiences and, when reported, outcomes.

**Method:**

Following PRISMA guidelines for scoping reviews, database searches (Scopus, Web of Science, ACM Digital Library, IEEE Xplore, ERIC, PsycINFO) were conducted in August 2025 for peer-reviewed English-language studies (2010–2025) in educational settings involving SPAs with social or relational capabilities and indicators of relationship quality. We excluded studies on physical robots, noneducational contexts, purely functional outcomes, or nonempirical/grey literature. After screening 216 unique records, 29 studies were retained and thematically analyzed to identify recurring design levers, relational constructs, and links to learning.

**Results:**

Studies showed substantial terminological and methodological diversity, with short, single-session experiments predominating. Eight recurring design levers shaping relationship quality emerged: (1) voice and appearance, (2) congruence and alignment, (3) empathy expression, (4) social safety and disclosure, (5) transparent personalization, (6) refutational explanations, (7) memory and consistency, and (8) interaction duration. Across studies, SPAs enhanced four key aspects of relationship quality: (a) social presence, (b) affective support, (c) trust, and (d) rapport. Learning gains were strongest when these indicators aligned with sound pedagogy.

**Discussion:**

Synthetic relationships with SPAs function as enabling conditions that enhance the learning climate—motivation, effort, and honesty—rather than directly improving test scores. We group the eight design levers into four categories: expressive (how the agent signals), relational (how it connects), transparency (what it communicates and why), and structural (how interaction unfolds). These, paired with the four relational indicators, form a practical framework for optimizing student–SPA relationships. Future work should prioritize multi-session and longitudinal designs, standardize relational measures, and address ethical concerns around purpose, transparency, influence limits, and shared oversight. When coupled with sound pedagogy, SPAs show strong potential to augment teaching and enrich learning.

## 1 Introduction

### 1.1 AI in education is evolving

The landscape of education is undergoing a profound transformation as artificial intelligence (AI) becomes increasingly integrated into learning environments. Unlike traditional educational technologies that primarily served as passive tools for information delivery, contemporary AI systems are evolving into sophisticated interactive partners capable of engaging in complex social behaviors (Guilherme, 2019). This shift represents more than a technological advancement; it marks the emergence of a new social context where learners interact with AI systems that can recognize emotions, respond empathetically, and adapt their behavior based on ongoing relationships (Kirk et al., 2025; Krämer et al., 2018).

Recent developments in artificial intelligence have enabled the creation of systems that can engage in sustained, meaningful interactions with students over extended periods (Zhou et al., 2024). These systems go beyond simple question-and-answer formats to participate in nuanced conversations, provide emotional support, and demonstrate social intelligence that increasingly resembles human-like interaction patterns (Gweon et al., 2023). As educational institutions worldwide integrate these technologies into their pedagogical frameworks, a new paradigm is emerging where learning occurs not just through human-to-human or human-to-content interactions, but through ongoing relationships between students and socially interactive AI systems (Cingillioglu et al., 2024; Loveys et al., 2022).

### 1.2 Defining socially interactive pedagogical AI systems

Socially interactive pedagogical AI systems can do more than provide individualized instruction, answer questions and give feedback; they can guide learners through problem-solving steps, adapt their explanations to a student’s needs, and offer hints or encouragement (Lippert et al., 2020; Schlimbach and Spill, 2023). The traditional terminology surrounding AI educational tools has been fragmented, with researchers employing various terms such as chatbots, virtual assistants, conversational agents, and embodied conversational agents (ECAs) inconsistently (Lugrin et al., 2021). Within educational contexts, terms like pedagogical agent or virtual tutor are also common. Lugrin et al. (2021) define socially interactive agents (SIAs) as virtually or physically embodied agents that are capable of autonomously communicating with people (and each other) in a socially intelligent manner using multimodal behaviors. Within this terminology, SIA serves as the umbrella category for both virtual embodiment (intelligent virtual agent) and robot embodiment (social robot). While the definition of SIA closely fits the underlying socio-relational mechanisms this review aims to explore, it systematically excludes text-only or audio-only systems that can still display social and emotional responsiveness relevant to our synthesis of evidence regarding social AI within an educational context.

In order to include social AI systems—with minimal or no embodiment—and to avoid confusion, we adopt a separate umbrella term: Social Pedagogical Agents (SPAs). While closely related to SIAs, these SPAs refer to educational AI systems that can exhibit meaningful social or relational interactions across different modalities (e.g., text-only, voice-based agents, and embodied virtual agents). Across these modalities, SPAs are characterized by three critical capabilities: (1) social cognition—the ability to interpret and respond to social cues, emotional states, and interpersonal dynamics; (2) adaptive interaction—the capacity to modify behavior based on ongoing relationship dynamics and user characteristics; and (3) presence—via visual, auditory, or textual manifestations that create a sense of agency and personality.

SPAs differ from previous educational technologies in their capacity for socio-affective alignment; the ability to participate in the social and psychological ecosystem co-created with users, where preferences and perceptions evolve through mutual influence (Kirk et al., 2025). This capability can in some cases enable them to establish what users perceive as genuine relationships, characterized by emotional connection, trust, and ongoing social engagement rather than merely functional interaction (Hu et al., 2025). The question remains if this evidence is just anecdotal or indicative of broader human-AI interaction, and whether such perceived relationships translate into benefits in educational settings.

### 1.3 The role of student-teacher relationships in education

Extensive research in educational psychology has consistently demonstrated that the quality of interpersonal relationships within learning environments fundamentally shapes educational outcomes (Roorda et al., 2011; Tao et al., 2022). The teacher-student relationship, in particular, has been identified as one of the most powerful predictors of academic achievement, motivation, and social–emotional development (Emslander et al., 2025; Allen et al., 2006). Students who experience positive, supportive relationships with their teachers demonstrate higher levels of engagement, improved self-efficacy, and better academic performance across diverse educational contexts (Chen and Huang, 2024; Mastul, 2024).

The mechanisms through which these relationships influence learning are multifaceted. Positive teacher-student relationships create psychological safety that enables students to take intellectual risks, express confusion, and engage in the vulnerable process of learning (Chacon Valdez et al., 2024). They provide emotional support that helps students regulate their emotions and maintain motivation during challenging academic tasks (Dai, 2024). Furthermore, these relationships serve as conduits for personalized feedback, mentorship, and the development of metacognitive skills that are essential for lifelong learning (Frisby and Martin, 2010).

As SPAs become capable of simulating many of the social and emotional behaviors that characterize effective human teaching, questions arise about whether similar relational dynamics can emerge between students and Social Pedagogical Agents. Preliminary evidence suggests that students can indeed form meaningful connections with SPA tutors and educational companions, leading to improved engagement and learning outcomes (Guo and Goh, 2015; Lou and Yang, 2024). However, the mechanisms underlying these relationships and their long-term educational implications remain underexplored.

### 1.4 Underexplored areas

Despite the growing interest in AI applications for education, several critical gaps exist in our understanding of how SPAs form relationships with students and how these relationships impact learning. Recent systematic reviews have identified limitations in the current literature that hinder our ability to develop evidence-based guidelines for implementing SPAs in educational settings.

Unfortunately, the field suffers from substantial terminological fragmentation and methodological inconsistency (Zawacki-Richter et al., 2019; Bond et al., 2024). Researchers use diverse terms to describe similar SPAs and employ varying theoretical frameworks to understand human-SPA relationships, making it difficult to synthesize findings across studies. This fragmentation has been exacerbated by the rapid technological evolution in the field, where new SPA capabilities outpace the development of standardized terminology (Hwang et al., 2020).

Additionally, there is a notable absence of longitudinal research that examines how student-SPA relationships develop over time and how they influence long-term educational and psychological outcomes (Ahmad et al., 2022). Most studies rely on short-term laboratory experiments or single-session interactions that cannot capture the dynamic, evolving nature of meaningful relationships. This limitation is particularly concerning given evidence from human relationship research suggesting that the benefits of positive teacher-student relationships accumulate over time through repeated positive interactions (Christophel and Gorham, 1995).

Most existing research has focused on functional outcomes—such as learning efficiency, content delivery, and immediate task performance—while paying insufficient attention to the relational and emotional dimensions of human-SPA interaction in educational contexts (Guilherme, 2019; Holstein et al., 2020). The few studies that do examine relationship quality often use inconsistent measurement approaches and theoretical frameworks, limiting our ability to understand the core mechanisms through which SPAs influence student experience (Winkler and Söllner, 2018).

Taken together, terminological fragmentation and the scarcity of longitudinal work help to explain why findings remain difficult to compare and why cumulative knowledge is slow to emerge. Yet in this article we do not set out to resolve those two structural issues. Our primary concern is the third gap: the limited attention to the relational dimensions of student-SPA interaction. We argue that, for SPAs deployed in real educational settings, synthetic relationships will play a key role in the learning process.

### 1.5 Research scope and questions

This study is guided by the need to establish a solid empirical foundation for understanding how emotional connections and synthetic relationships form between students and SPAs in educational settings, what factors influence their quality, and how they impact educational outcomes. Unlike previous reviews that have generally examined SPA in education broadly, our focus is specifically on the relational and emotional dimensions of student-SPA interaction within educational contexts. We include studies that examine various forms of Social Pedagogical Agents, provided they demonstrate capabilities for social interaction and relationship formation with students. This systematic review is structured around one central research question: *“How do design features and interaction characteristics facilitate the development of synthetic relationships - between students and Social Pedagogical Agents - that promote students’ learning outcomes?”*

By addressing this question, this review aims to provide educational researchers and practitioners with a comprehensive understanding of the existing evidence base while highlighting key priorities for future research and development. Furthermore, by identifying the effect of design features and interaction characteristics on relationship quality indicators, we aim to describe their indirect effect on the learning experience. These insights will inform the development of a practical guide for the implementation of Social Pedagogical Agents within an educational context, including specific recommendations to improve learning outcomes through effective use.

## 2 Methods

**Figure 1:**
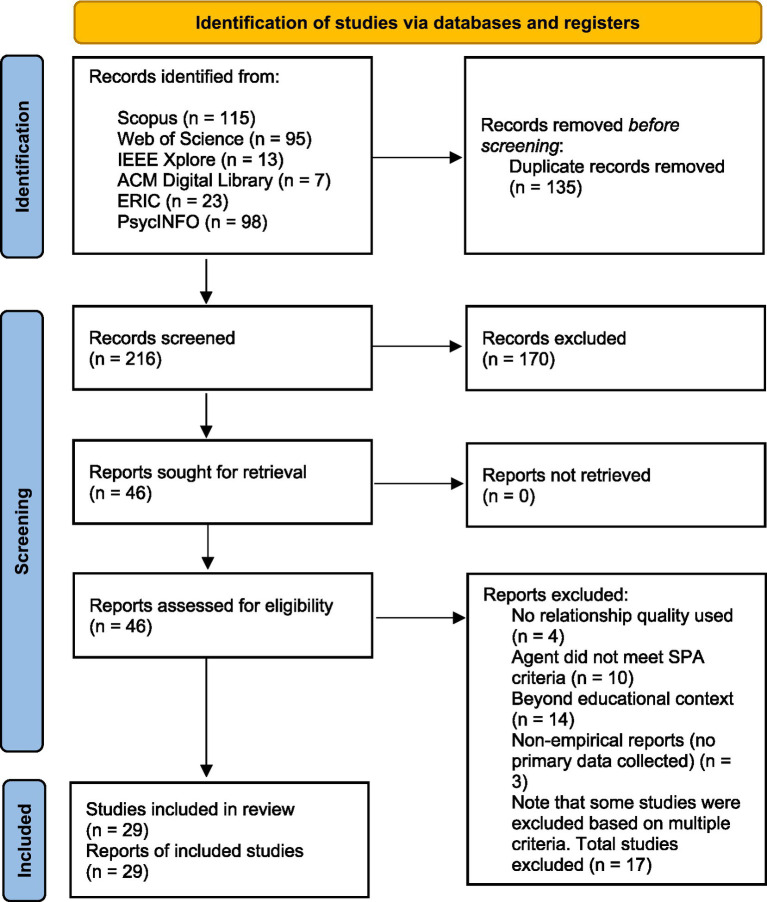
PRISMA flow diagram adapted from PRISMA 2020 (Page et al., 2021). The figure provides a visual summary of the study identification, screening, and inclusion process.

This scoping review was conducted in accordance with the PRISMA 2020 framework (Page et al., 2021). PRISMA guidance informed the full study selection workflow, including the eligibility criteria, search strategy, identification process, screening process, and final inclusion decision. The number of records at each stage is documented in the PRISMA flow diagram (Figure 1).

### 2.1 Eligibility criteria

Eligible studies were peer-reviewed, empirical investigations published in English after January 2010. Studies prior to 2010 frequently reflect earlier technological limitations in natural language processing, graphical realism, and AI-driven interaction capabilities, potentially reducing comparability with current systems and limiting their relevance for contemporary assessments of relationship quality. Accepted study designs included randomized controlled trials, experimental, quasi-experimental, observational, and survey studies that gathered primary data and were published in peer-reviewed journals. While high-quality research is often published in conference proceedings, journal articles typically provide more comprehensive reporting and undergo more standardized review processes.

Because several educational systems deliver relational behavior through text-only or minimally embodied interfaces, we included text-based agents when the study demonstrated social or emotional responsiveness (e.g., empathy, self-disclosure, name-based personalization) in an educational task. We excluded purely informational chatbots, because these do not support the kind of relationship-quality indicators under review. Eligible studies examined learners in educational settings interacting with Social Pedagogical Agents (SPAs) that (i) displayed presence—whether through visual, auditory, or textual cues—that create a sense of agency and personality, (ii) supported natural-language conversation, and (iii) displayed social or emotional responsiveness. To be included, studies reported at least one indicator of relationship quality—such as rapport, trust, social presence, connectedness, empathy, or closely related constructs.

Studies were excluded if they met any of the following criteria: the study focused physically embodied robots; the study assessed only functional outcomes without relational measures; the study was conducted outside educational contexts; the type of social connection examined in the study was not relevant to our review (e.g., studies focusing solely on user satisfaction or system efficiency, without addressing connectedness); or the study comprised non-empirical or non-peer-reviewed formats (e.g., conference proceedings, dissertations, book chapters, grey literature, theoretical reviews, or position papers).

### 2.2 Search strategy

To ensure comprehensive coverage of the interdisciplinary literature spanning education, computer science, human-computer interaction, and psychology, we conducted systematic searches across six major databases: Scopus, Web of Science, ACM Digital Library, IEEE Xplore, ERIC and PsycINFO. This combination of education-, HCI-, and AI-oriented databases was chosen to capture work on Social Pedagogical Agents that is often published outside education-only venues. The search strategy was developed through an iterative process involving (1) initial scoping searches to identify key terminology, (2) consulting existing systematic reviews and the handbook on socially interactive agents (Lugrin et al., 2021), (3) testing the search strings to balance sensitivity and specificity, and (4) refinements based on preliminary results. The final search string combined three concept blocks with Boolean AND; synonyms within blocks were combined with OR. Exact syntax was adapted to each database, but the logical structure remained constant.

*Relationship Quality Indicators:* rapport OR connectedness OR closeness OR attachment OR “social presence” OR trust OR empathy OR “emotional connection” OR “social bond” OR immediacy.

*Agent Terminology:* “conversational agent*” OR “embodied conversational agent*” OR “social agent*” OR “relational agent*” OR “virtual agent*” OR “intelligent virtual agent*” OR “virtual human*” OR “digital human*” OR “pedagogical agent*” OR “socially interactive agent*” OR “socially intelligent agent*” OR “virtual tutor*.”

*Educational Context:* education OR educational OR teacher* OR student* OR learner* OR classroom OR “learning environment*” OR pedagog*.

### 2.3 Selection process

Searches were conducted in August 2025, with date limits set from January 2010 to August 2025. The screening process is described in Figure 1, consistent with PRISMA 2020 reporting standards (Page et al., 2021) and the articles selected for analysis are listed in Appendix 1.

Following PRISMA’s guidelines, records were first screened by title, to exclude clearly irrelevant studies, with any uncertainties discussed and resolved collaboratively. Subsequently, titles and abstracts were assessed independently by two reviewers against the eligibility criteria (i.e., studies set in an educational context, using a SPA with social or relational aspects, and including a measure of relationship quality). Inter-rater agreement for this stage was moderate (Cohen’s *κ* = 0.53). Remaining discrepancies were addressed through further review at the full-text level to determine whether studies met the inclusion criteria.

### 2.4 Data extraction and synthesis

For each included study, we extracted key information: the agent’s characteristics (embodiment, modality of communication, any affective or adaptive features), the study design (sample size, context, duration of interaction, and experimental manipulations, where applicable), the relationship measures used (affection, rapport, trust, etc.), outcome measures (learning gains, motivation, etc.), and main findings relevant to relationship quality and learning outcomes. Given the heterogeneity of SPA and relational terminology, and the diversity of measurement scales, a quantitative meta-analysis was not defensible; we therefore used thematic synthesis. We grouped findings inductively into thematic categories reflecting how design features and interaction characteristics influence synthetic relationships and how categories of synthetic relationship indicators influence learning outcomes. The discussion section will offer a consolidated perspective, design recommendations, and future research directions.

## 3 Results

Across the 29 included studies (2012–2025), spanning higher education, professional/medical training, and various subject domains, we synthesized how learners relate to Social Pedagogical Agents and what those relationships mean for learning.

### 3.1 Prevailing trends and patterns in terminology and methodology

#### 3.1.1 Relational constructs and measures

In line with our preliminary findings, described in the introduction, studies employed a diverse set of relationship quality indicators: trust/credibility (Chiou et al., 2020; Coleman et al., 2025; Ivanović et al., 2015; Laverde et al., 2025), rapport (Krämer et al., 2016; Cingillioglu et al., 2024), (emotional) connectedness (Savin-Baden et al., 2013), social presence (Eiris et al., 2021; Hong et al., 2024), and empathy/support (Guetterman et al., 2019; Hopman et al., 2023). Some studies even assessed these constructs in combination (e.g., rapport with social presence: Schmidt et al., 2024; empathy with trust: Kim et al., 2025; Ryan et al., 2025). Measurement relied largely on heterogeneous self-report scales, with occasional ad-hoc items (e.g., “perceived helpfulness/friendship”), limiting comparability. A striking omission across the studies we reviewed is the use of well-validated classroom relationship instruments from educational psychology, such as the Questionnaire on Teacher Interaction (Wubbels and Brekelmans, 2005). This model describes teacher–student relationships in terms of teacher behavior and could be adopted or adapted to describe SPA-student relationships in terms of SPA behavior. This would make results more comparable to earlier classroom research and would greatly enable cross-study and cross-paradigm comparison.

#### 3.1.2 Labeling heterogeneity of SPAs

As anticipated, terminology was broad and overlapping: pedagogical/virtual/embodied conversational agents, virtual tutors/coaches, and role-first labels were used interchangeably. Examples range from embodied virtual human (Eiris et al., 2021), to virtual tutor (Ding et al., 2023), and virtual patient (Pataki et al., 2012). These labels often masked comparable underlying capabilities, supporting our adoption of “Social Pedagogical Agents (SPAs)” as an umbrella term for these closely related social AI systems within an educational context.

#### 3.1.3 Methodological patterns

Echoing our introduction, the literature is dominated by short, controlled exposures (typically 15–60 min) with between-subjects manipulations (e.g., empathic vs. neutral demeanor; human-like vs. synthetic voice) and immediate post-session outcomes (social presence, trust, engagement, short-term retention). Only one study assessed effects on self-efficacy 2 weeks after the SPA-student interaction (Coleman et al., 2025). Field-based work was limited (e.g., Ding et al., 2023; Huang et al., 2024), with notable but usually one-off simulations in medical education (Laverde et al., 2025). Consequently, we know little about how SPA–student relationships evolve or endure, but there is a wealth of diverse information about initial effects of synthetic relationships between students and SPAs.

### 3.2 Design features and interaction characteristics that affect the development and quality of synthetic relationships

Across otherwise diverse settings and SPA implementations, we identified eight recurring design levers that affect relationship quality indicators across the studies. These design elements emerged as factors that, when manipulated or observed, had significant effects on how students related to the SPA. We present each in turn.

#### 3.2.1 Voice and appearance

Design attributes such as voice and appearance strongly influence first impressions—social presence, trust, immediacy—and can also moderate the effects of other instructional features. Voice quality, in particular, reliably altered perceptions of credibility and trust: in an online study with a virtual human tutor, high-quality TTS and especially a human voice were judged more credible and engaging than a low-quality TTS voice, with trust ratings highest for the human voice (Chiou et al., 2020). Expressive vocal delivery primarily strengthens immediacy—the sense of psychological closeness—which, in turn, elevates affective responses like liking and perceived rapport. In a controlled experiment, a SPA with strong nonverbal, vocal expressiveness (faster rate, higher pitch variation) increased perceived immediacy (Fountoukidou et al., 2022).

Appearance shows a similar pattern. In a VR storytelling study that manipulated narrator appearance fidelity (abstracted vs. modeled vs. realistic), students’ persona ratings (e.g., credibility, human-likeness, engaging) did not differ significantly across fidelity levels—suggesting that making a face more realistic does not, by itself, deepen the perceived relationship (Eiris et al., 2021). Nonetheless, the study noted that some relational responses (e.g., topic interest/engagement) varied with simple persona cues such as age and gender alignment, underscoring that appearance can still tune first-impression affinity when those cues match audience expectations. Giving users control over attributes such as gender, clothing or hair of a SPA, can significantly increase rapport, trust, and the perceived social presence of SPAs during subsequent interactions in virtual reality (Schmidt et al., 2024).

Social presence findings in public-speaking VR likewise emphasize reactions and perceived responsiveness over visual richness per se. Participants’ sense that the (virtual) audience reacted to them—more than co-presence or interface interactivity—was the social-presence dimension tied to their experience, highlighting that felt reciprocity is central to relational quality in these settings (Poeschl, 2017). It is important to note that this study used a scripted, non-contingent audience; what predicted participants’ experience was their own felt reaction to this audience and the scenario’s perceived responsiveness, not genuinely adaptive audience behavior. Sanjeewa et al. (2025) further showed that voice-only empathy was easy to detect at high or low levels but ambiguous at mid-levels, leading the authors to recommend multimodal cues for clearer relational signaling. When nonverbal cues such as nodding, smiling, or mutual gaze are coordinated with the verbal message, learners report higher rapport and social presence (Krämer et al., 2016).

#### 3.2.2 Congruence and alignment

Students respond not only to what the SPA says but to whether its demeanor matches its role and context. Fountoukidou et al. (2022) and Chiou et al. (2020) showed that strong vocal expressiveness promotes immediacy and trust, but Liao et al. (2024) clarified that this expressiveness must be role-congruent (e.g., peer vs. teacher vs. assessor). Liao et al. (2024) demonstrated interactive effects of SPA role and emotional expressiveness: in their experiment with children, a peer-like SPA with an enthusiastic, warm voice achieved higher engagement in a creative task, whereas a teacher-like SPA was more effective when using a structured, calm tone. Children expected a peer-role SPA to be friendly and energetic. When that expectation was met, they responded with greater interest. Conversely, a peer-role SPA using a stern teacher voice confused them. Similarly, Nebel et al. (2020) found that in an educational game, when the SPA was presented as a competitor peer, a more playful or challenging demeanor worked well, whereas a tutor SPA performing stepwise problem-solving benefited from a more neutral, authoritative style.

Role expectations also shape trust; Ivanović et al. (2015) used a tutor that randomly switched between helpful and misleading hints for students working on a code programming task. Their results showed that more experienced students saw through the deception, and in these cases, students were more motivated to knowingly use the deceptive SPA, but students who were inexperienced tended to trust the tutor far more. This implies that perceived trust does not always require actual trustworthiness and is at least partly dependent on expectations regarding the role and context in which a SPA is used. Students and teachers explicitly endorse a “good teacher” style that combines relational and task-related communication and argue that SPAs should shift between these modes as needed (Sikström et al., 2024).

Overall, these studies suggest practical guidance: match warmer, more expressive, rapport-rich behavior with peer, coach, or competitive-peer roles, and use more structured, calm, and authoritative behavior with teacher/tutor roles; ensure that all nonverbal signals support the same communicative intent; and avoid mismatches between what the SPA claims to be and how it behaves.

#### 3.2.3 Empathy expression

Moridis and Economides (2012) found that when a SPA synchronized empathetic messages with matching facial and vocal expressions, a sequence of “parallel” empathy (reflecting the learner’s emotion) followed by “reactive” empathy (aimed at shifting the emotion) was more effective in moving fearful learners toward neutrality than simple mirroring, which could inadvertently prolong negative affect. This shows how voice tone and facial affect can amplify perceived care and support when they are timed and targeted, rather than merely more “emotional.”

It is not just the congruent timing that is important, within the context of the interaction, responsiveness is also important. Affect-sensitive SPAs that detect and respond immediately to learners’ emotional states—heighten the student’s sense of support: Wang et al. (2025) found that a SPA which immediately gave a sympathetic acknowledgment when a student showed frustration (“Don’t worry, it’s normal to feel frustrated”) led to a stronger feeling of support, compared to a SPA that offered empathetic remarks only after a delay or only after the student failed.

#### 3.2.4 Social safety and disclosure

Pataki et al. (2012) observed a preference for rapport developing behavior over more task-relevant diagnostic questioning in an experiment where the SPA was a virtual adolescent patient being interviewed by students. This study shows how almost all 15 students spontaneously prioritized building rapport and alludes to a preference for establishing a safe environment before gathering detailed medical information. Cingillioglu et al. (2024) used an autonomous SPA, specifically with a non-intrusive stance, thereby allowing respondents to speak without interruptions. In order to encourage students to share their valuable insights, researchers strived for an environment where the SPA made students feel at ease and fostered rapport and confidence. After completing the experiment, students voluntarily offered appreciative closings (e.g., “this was a really fun,” “Great AI tool, I love the way it calls me by my name, it makes it feel personalized”), continued the conversation and finally ended the chat as if they were talking to another human. This was interpreted as a sign that the SPA had built a strong rapport with participants.

Relatedly, self-disclosure by a pedagogical SPA increased students’ perceived intimacy and cognitive trust, which improved acceptance of feedback, especially when feedback was negative (Kim et al., 2025).

Taken together, these findings suggest that, in sensitive domains requiring student vulnerability, establishing social safety via a stable, non-judgmental persona and clear empathic cues can increase both engagement and disclosure.

#### 3.2.5 Transparent personalization

SPAs that adapt their feedback or dialog to a learner’s individual goals, knowledge, or emotional state and, crucially, that explain the rationale behind their responses, increased students’ sense of support and acceptance of the SPA’s suggestions. In a study by Nelekar et al. (2022), a stress-management virtual advisor offered study tips that were personalized (based on each student’s stated beliefs or goals) and provided brief explanations (“I’m suggesting this because you said X”), fostering higher trust and a stronger working alliance between students and the system. Importantly, personalization was done transparently: if a SPA adapts without explaining itself, students can perceive its behavior as arbitrary or unfair, potentially eroding trust. Conversely, even lightweight transparency—a simple note explaining why a piece of advice is given, referring to data about the user—is sufficient to increase user acceptance of the SPA’s assistance (Nelekar et al., 2022). Huang et al. (2024) also employed a SPA that did not just answer questions, but gave individual users targeted situation-specific feedback. This personalized feedback affected students’ perception of the SPA, making it appear more competent. Additionally, students found the SPA’s feedback very helpful. In summary, this lever suggests that students form better relationships with SPAs that treat them *as individuals* and reveal their reasoning. Personalization that is visible and understandable enhances the SPA’s perceived benevolence and competence, key components of trust.

#### 3.2.6 Refutational explanations

When Schroeder et al. (2023) used a SPA to teach about genetically modified foods, they found that when the SPA confronted students’ misconceptions using a refutation narrative structure (explicitly acknowledging a student’s incorrect belief and then correcting it), learners did not report higher credibility of and trust in the SPA, compared to when the SPA provided purely expository explanations without refutation. Also, message trust (learners’ confidence that the specific information delivered by the SPA was accurate) emerged as the critical predictor of learning gains, indicating that acceptance of the SPA’s corrective content is a key mechanism through which SPAs can influence learning. It would seem that transparent SPAs that make the basis, timing, or intent of their responses more explicit, are better positioned to secure this message-level trust.

Alternatively, Ding et al. (2023) showed that students in a physics class can trust a SPA even when its answers are not accurate. They even found that a higher level of trust was associated with a higher accuracy assessment of (incorrect) answers. So, while Schroeder et al. (2023) found that message trust was more important than trust in the SPA, Ding et al. (2023) found that trust in the SPA can increase trust in the message. This blind faith that Ding et al. (2023) found further reinforces the suggestion that transparency is important to assess if information is correct to avoid students relying on trust. Similarly, Sikström et al. (2024) found indications that students have higher trust in an answer from a human teacher than a SPA and they recommend increasing predictability, transparency and explainability as ways to enhance trust in SPAs.

#### 3.2.7 Memory and consistency

As SPAs become more common, the way a SPA uses memory (i.e., what it remembers from the ongoing interaction and provided information) can shape relational perceptions. In a clinical training context, Laverde et al. (2025) used a virtual patient and found that configuring the SPA with a bounded memory (limited to the current patient case) achieved coherent, on-topic dialogues and high usability ratings. Essentially, the SPA was prevented from bringing up irrelevant past cases or inconsistencies; its dialogue capabilities were restricted by what a given patient should know in that scenario. This bounded consistency in the SPA’s factual memory increased perceptions of trustworthiness; the SPA appeared reliable and realistic because it did not contradict itself, make up facts, or act forgetful within the scenario.

In sensitive settings, students reported feeling more comfortable and truthful when a pedagogical SPA did not recall prior personal conversations beyond the immediate task (i.e., when it lacked persistent memory across contexts), highlighting that limited or non-persistent memory can be beneficial to disclosure and comfort (Savin-Baden et al., 2013).

#### 3.2.8 Interaction duration

Finally, although not a design feature per se, the duration and continuity of interaction seem to be a logical feature to enhance relationship quality. Across our corpus, short single-session interactions (often under an hour) reliably produced shifts in proximal social outcomes. For example, single brief exposures to a virtual human changed learners’ trust (Chiou et al., 2020), a 15-min coaching session produced high working-alliance ratings (Hopman et al., 2023), a 10- to 15-min educational interaction increased trust and satisfaction (Coleman et al., 2025), and a single 20-min competitive-SPA session increased behavioral engagement and elevated facets of social presence (Nebel et al., 2020), to name but a few. However, truly durable bonds would plausibly require a SPA that remembers prior encounters, shares a history with the learner, and adapts over time; features that were rarely evaluated in our set. Most studies in the corpus were similar single-session interactions with only a few exceptions. One notable study, conducted by Huang et al. (2022) over a nine-day period, showed how participants perceived the SPA’s social presence and experienced interpersonal attraction to the SPA. A 12-week chatbot-supported course showed how a SPA, available 24/7, can create a sense of emotional engagement (Huang et al., 2024).

Unfortunately, while both examples of long-term interaction show increased relationship quality, they never directly compare the effect of multi-session or multi-week relationship building to shorter single-session interactions; leaving us to ponder if truly durable bonds require a SPA to remember prior encounters, share a history with the learner, and visibly adapt over time. Duration could be a potential lever: short sessions *can* shift trust, and rapport, but extended interaction is hypothesized to be necessary for true relationship development; analogous to how human teacher-student rapport builds over a semester.

Taken together, appearance and voice chiefly moderate relationship quality by shaping trust, immediacy, and perceived responsiveness. Appearance matters most when persona cues (e.g., age/gender) align with user expectations, even though realism alone rarely deepens the bond. Human-like or high-quality voices tend to boost trust and credibility and expressive delivery fosters immediacy and liking. Specifically, the empathic alignment of voice and facial cues can convey care and support. These eight design elements provide a practical lens for understanding what makes an educational SPA more or less “relatable” and effective in the eyes of students. This leads us to our next question: if a synthetic relationship between SPA and learner can be formed, how does this influence learning outcomes?

### 3.3 How synthetic relationships affect learning experience and learning outcomes

Data were analyzed to show how specific aspects of the student-SPA relationship (social presence, affective support, trust, empathy and rapport) affect learners’ experiences and learning outcomes. In general, positive relationship indicators tended to function as enabling factors: higher trust, rapport, or presence typically led to more engagement and better uptake of the SPA’s instruction, which in turn could improve learning. However, not all relationship aspects translated equally into measurable learning gains, and some primarily influenced affective-motivational outcomes. We organize this section by key relational constructs.

#### 3.3.1 Social presence

A sense of connection with a SPA can lead to beneficial behavioral learning outcomes. This is true for both rapport and social presence. Studies show that rapport (Krämer et al., 2016), social presence (Hong et al., 2024), immediacy (Fountoukidou et al., 2022), and emotional engagement have the potential to result in better performance. Fountoukidou et al. (2022) also noted that students taught by an expressive-voice SPA reported higher perceived cognitive learning (they felt they learned more) and showed greater affective learning, even though actual test scores were only slightly higher compared to a dull-voice SPA. Chiou et al. (2020) found that modern text-to-speech and human voices produced different levels of trust and perceived qualities of the virtual human, while learning outcomes were not reliably affected. In the same vein, when voice “quality” differed, learners’ trust changed even if their performance did not, indicating that instructional impact can remain stable while the experience of learning improves.

In an English-learning “Charades” chatbot, social presence positively related to hedonic and epistemic value and, in turn, to behavioral learning outcomes (Hong et al., 2024). When students felt a SPA was more “present” and interactive, they treated it more like a social partner and invested more effort (for example, practicing conversation longer because it felt engaging). Learners who experienced a stronger sense of “being with” the chatbot reported higher value and achieved better performance on a vocabulary test within that design.

Social immediacy cues can further support engagement and aspects of learning. For example, a SPA with strong vocal expressiveness increased perceived nonverbal immediacy and enhanced affective and perceived cognitive learning, with motivation partly mediating these effects (Fountoukidou et al., 2022). Chiou et al. (2020) showed that SPA voice quality may not directly change test performance, but it still shaped learners’ judgments about the SPA, which is relevant for whether learners continue to learn from it.

Another experiment by Poeschl (2017) in public speaking training compared a richer communicative bandwidth (VR with avatar audience providing nods and facial reactions) vs. a more minimal interface; students in the richer, more socially present condition showed improved engagement and even better performance in subsequent speeches. Appearance-related manipulations showed something similar. In the VR storytelling study by Eiris et al. (2021), higher-fidelity avatars increased perceived credibility and social presence, and students reported increased topic engagement; however, learning outcomes did not significantly differ between high- and low-fidelity conditions in that study, and the gains were context-dependent and only modestly affected by appearance realism.

Savin-Baden et al. (2013) provided qualitative evidence that when students felt “it’s almost like talking to a person,” they were comfortable disclosing truthful and detailed answers about sensitive topics to the SPA. The SPA’s persona (its portrayed character or role) and communicative stance significantly influence how safe and willing students feel to engage and disclose. When participants experienced a greater sense of social presence, they were more willing to disclose and provide truthful answers on a sensitive questionnaire, because the SPA’s presence feels less judgmental than a human teacher’s. This openness is described as “genuine emotional engagement,” and can be crucial in tutoring scenarios too. If a student openly tells a SPA “I do not understand this,” the SPA can adapt and clarify, whereas if the student stays silent, a learning opportunity is missed.

Taken together, these findings support the claim that social connections with SPAs function as enabling conditions: they encourage behaviors that can be directly facilitate learning and improve results.

#### 3.3.2 Affective support

Empathic behavior by the SPA generally improved students’ learning experience and affective learning outcomes, such as motivation, self-efficacy (confidence in learning), emotional state, as well as a cognitive outcome (knowledge retention) and behavioral outcomes (persistence); all of which are important precursors to learning. Hopman et al. (2023) noted that their empathetic “digital coach” ERICA successfully built an alliance with students and those with stronger alliance were more likely to express intention to use their newly learned strategies. Ryan et al. (2025) suggest that the specific design of a SPA, that incorporates interactive and practice-oriented elements in a controlled virtual environment, is a feasible approach to address self-efficacy barriers. A safe practice environment, where learners do not feel judged, removed obstacles to effective learning through increased self-efficacy and confidence of learners.

Oker et al. (2020) discovered that a bimodal SPA, that combined its verbal feedback with facial expressions, was perceived as more empathic. Additionally, interactions with the bimodal SPA correlated positively with both accuracy and more correct answers. The authors attribute the difference to increased empathy and motivation. Similar findings by Moridis and Economides (2012) underscore that empathy intended to regulate (not only mirror) emotions may be beneficial for learning. They found that a SPA that expressed empathy, while displaying emotional facial expressions congruent with the tone of voice, improved learners’ emotional state from fear to neutral.

Notably, Krämer et al. (2016) found that empathy in SPAs primarily moved *motivational* outcomes; learners felt more supported, invested more effort, and had better emotional regulation. More fine-grained designs indicate *how* empathy matters for learning outcomes. For instance, Wang et al. (2025) explicitly found that a SPA providing parallel empathy (immediate supportive responses to student emotions) made students feel more supported and contributed to them spending more time on tasks, which then led to higher quiz scores in that condition. Additionally, reactive empathy (support that aims to change the learner’s emotion) enhanced attention to key content and improved retention and transfer; providing both parallel empathy (immediate supportive mirroring) and reactive empathy yielded the strongest transfer performance.

Taken together, these studies indicate that empathy primarily moves motivational and affective outcomes: learners feel more supported, invest more effort, and regulate emotions more effectively (Moridis and Economides, 2012; Oker et al., 2020). However, empathy alone does not guarantee higher test scores; benefits for learning are clearest when affective support is paired with sound instructional design and scaffolding, consistent with frameworks that position empathy as a driver of social–motivational processes that enable (rather than directly cause) learning (Wang et al., 2025).

#### 3.3.3 Trust

Learners’ trust in a SPA emerged as important for how students respond to the SPA’s guidance. Across several studies, higher perceived trust or credibility were associated with students’ willingness to follow the SPA’s guidance and to engage more deeply with the material. For example, Chiou et al. (2020) showed that when a virtual tutor used a higher-quality (or human) voice, students reported greater trust and credibility. Although voice quality did not significantly affect learning outcomes, their results do suggest trust may contribute to learning as it makes the SPA more credible and engaging. Ivanović et al. (2015) experimented with SPAs that deliberately gave misleading hints, and found that inexperienced students trusted the SPA blindly, but the more experienced students, who noticed the tutor’s inconsistent help, were more motivated to use the SPA and employ critical thinking during the assignments, indicating that the way the credibility of the SPA is perceived can impact the depth of engagement with the learning task.

Disclosure is a relevant mechanism to foster trust. As shown in Section 3.2.4, when pedagogical SPAs offered brief, task-relevant self-disclosures, students reported greater intimacy and cognitive trust; cognitive trust also mediated higher acceptance of the SPA’s feedback—effects that were especially pronounced after negative feedback (Kim et al., 2025). Such disclosure can help sustain rapport and keep interactions productive following setbacks.

Another study on a chatbot tutor cautions that trust alone is insufficient. Ding et al. (2023) reported that students’ perceived credibility of ChatGPT-as-tutor was positively correlated with how much they said they learned and their intention to keep using it. However, trust did not guarantee learning improvements if the SPA’s content was poor; for example, if a SPA was likable and trusted but gave incorrect hints, it could mislead students. Trust, as Schroeder et al. (2023) point out, is not just a single thing: it can refer to a broader, evolving relationship with a virtual human, while credibility is a more targeted judgment about the quality of what the SPA says. In their science-tutoring study, they intentionally measured both levels—trust in the SPA and trust/credibility in the message itself—and found that these constructs were correlated but still empirically distinct. Crucially, only message trust (i.e., believing the information was accurate and reliable) was significantly associated with learning outcomes; trusting the SPA as a character was not. The takeaway they emphasize is design-oriented: if the goal is to support learning, designers should prioritize features that increase credibility and trust in the instructional message itself (clear, accurate, well-supported explanations) because that was the piece that actually predicted learning, whereas making the SPA more “trustworthy” as a social partner did not show the same payoff. Once learners believe the message, they are more likely to use what the system offers (e.g., accept the explanation), which in turn supports better performance.

In summary, trust/credibility is an essential enabler: it can significantly amplify or dampen the instructional effectiveness of a SPA. It stands to reason that any design choices undermining credibility (like the SPA making factual errors or behaving inconsistently) may harm the learning experience via loss of trust. However, the importance of trust and credibility should not be overstated. It is important for effective SPA-supported instruction, but its impact on learning depends on the accuracy and believability of the SPA’s messages: increasing trust in what is said (not just who says it) is most directly linked to learning.

#### 3.3.4 Rapport

When comparing a SPA that displayed rapport (e.g., smiling and nodding at the right moment) behavior versus one that did not, Krämer et al. (2016) found that rapport behavior was able to foster both better performance and more effort (but not motivation) from students. They conclude that their SPA that showed rapport behavior had a similar positive effect on performance as with students engaging face-to-face with human instructors. Notably, these effects appeared even when students’ *self-reported* sense of rapport did not increase, suggesting that rapport behaviors can be effective even when it is not noticeable. In a study by Cingillioglu et al. (2024), participants responded with richer qualitative insights to open-ended questions asked by a SPA. These insightful, authentic and accurate answers, improved reliability and validity of the data and were attributed by the researchers to the strong rapport between SPA and participants.

## 4 Discussion

### 4.1 Effect of relationship quality on learning experience and learning results

This review set out to synthesize how “synthetic relationships” with Social Pedagogical Agents are formed and sustained in educational contexts, and how those relationships shape learners’ experiences and outcomes. First, we identify four main categories of design levers that have shown to be instrumental in forming and maintaining synthetic relationships: epistemic, relational, expressive, and structural levers are identified as design variables to enhance relationship quality. Second, using these four levers, SPAs show the ability to evoke social and relational responses in learners: trust/credibility, rapport, social presence, and affective support are routinely identified. However, these relational variables do not always directly lead to better learning results; they can function as enablers. These relational outcomes create a context where students’ learning experience is enhanced. To achieve improvements in learning results, the learning experience can be paired with strong instructional scaffolding (e.g., targeted refutations of misconceptions, well-timed feedback). In other words, relational quality is not always enough in and of itself but sometimes is a means for SPAs to contribute to sound pedagogy and accurate content. This framing clarifies apparently mixed results in the literature: a warm, credible, present SPA cannot compensate for incorrect or poorly sequenced instruction, yet when the instruction is solid, the same relational qualities often unlock fuller engagement and better uptake.

### 4.2 Impact of design levers on learning experience through increased relationship quality

Based on our analysis we distilled the design features and interaction characteristics into four distinct levers. These design levers directly impacted relationship quality, which had an impact on students’ learning experience. Transparent adaptation levers describe how the SPA adapts to a user and why. This includes refutational explanations and transparent adaptability. Relational levers capture how the SPA connects; stances and behaviors that foster social safety and disclosure, empathy and encouragement, and role-appropriate demeanor. Expressive levers cover how the SPA expresses messages; voice, gaze, gesture, and other modalities that are congruent with each other and that align these signals with intent. Structural levers describe how the interaction unfolds over time; this includes continuity, memory consistency, and session scaffolding that make growth visible.

#### 4.2.1 Expressive levers

Multimodal alignment (e.g., synchronizing gaze, gesture, facial affect, and prosody with the message) consistently improved social presence and rapport beyond appearance realism alone (Krämer et al., 2016; Moridis and Economides, 2012). Voice quality and user-tunable appearance shaped first-impression trust and immediacy; expressive prosody increased perceived learning and liking even when scores moved modestly (Chiou et al., 2020; Fountoukidou et al., 2022; Eiris et al., 2021; Schmidt et al., 2024). Across studies, “more realism” was neither necessary nor sufficient and coherent signals mattered more than fidelity.

#### 4.2.2 Relational levers

Two complementary relational levers emerged: empathy and encouragement, and social safety/ disclosure. Immediate parallel empathy validates feelings; reactive empathy then helps regulate emotions and steers toward task focus, which yielded better persistence and transfer than mirroring alone (Moridis and Economides, 2012; Wang et al., 2025; Oker et al., 2020). Social safety (e.g., a non-judgmental stance, options to opt-out, respectful closings, calibrated self-disclosure) was associated with greater disclosure and willingness to ask for help (Savin-Baden et al., 2013; Kim et al., 2025; Ryan et al., 2025; Cingillioglu et al., 2024). Finally, role–behavior congruence mattered: peer/coach voices benefited from warmth and enthusiasm; tutor/assessor roles from calm structure (Liao et al., 2024; Nebel et al., 2020; Krämer et al., 2016). Incongruent styles produced confusion (Ivanović et al., 2015).

#### 4.2.3 Transparency levers

Two design features emerged as consistently consequential for relationship quality and learning enablement: refutational explanations for common misconceptions, and transparent personalization that makes the SPA’s reasons legible (e.g., “I’m suggesting X because you said Y”). Refutational tutoring increased credibility and conceptual change on misconception-dense topics, suggesting that epistemic candor (acknowledging the learner’s belief and then correcting it with evidence) boosts message trust—the belief that the content is accurate—more reliably than generic expository feedback (Schroeder et al., 2023). It is important to note the distinction between message credibility (epistemic trust) and SPA credibility (persona trust). Message credibility is directly linked to learning outcomes, while SPA credibility helps primarily by increasing compliance with guidance (Schroeder et al., 2023; Ding et al., 2023). Transparent personalization likewise increased perceived benevolence and acceptance of guidance; small “why you see this” rationales were often sufficient to reduce arbitrariness and enhance cooperation (Nelekar et al., 2022; Nebel et al., 2020).

#### 4.2.4 Structural levers

Two pivotal structural levers are interaction duration and memory and consistency. Even brief exposures shifted proximal social outcomes, yet durable bonds likely require visible growth across encounters (Chiou et al., 2020; Hopman et al., 2023; Nebel et al., 2020). Multi-session deployments reported strengthened social presence and engagement over time (Huang et al., 2022, 2024), though direct contrasts with single-session baselines remain rare. Memory policy influenced comfort and credibility: bounded, auditable memory (case-scoped or domain-scoped) supported coherence and realism without over-personal retention, which some learners preferred for sensitive tasks (Laverde et al., 2025; Savin-Baden et al., 2013).

#### 4.2.5 From SPA features to learning outcomes

**Figure 2:**
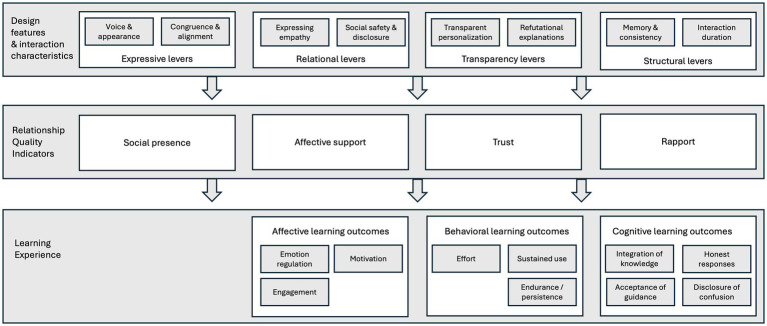
How design features and interaction characteristics affect learning experience through synthetic relationships.

SPAs do not improve learning simply by “being social,” but because specific design features make certain relationship indicators more likely, and those indicators in turn enable better learning experiences and, under sound pedagogy, better learning results. Figure 2 links four design levers we distilled from the corpus (expressive, relational, transparency and structural levers) to the relational states they most often activated in students (social presence, affective support, trust and rapport) and finally to the specific effects on the learning experience (classified as affective, behavioral and cognitive learning outcomes). In doing so, it makes explicit that synthetic relationships are best understood as mediating conditions that enable learning, rather than as learning outcomes in their own right.

In order to provide practical guidelines for designers and educators, Table 1 translates the pathway from Figure 2 into an actionable format. For each of the four design levers, it specifies (a) what the SPA should actually do in interaction, (b) which relationship indicator this is most likely to strengthen or what relational pitfall it will avoid, and (c) what kind of learning-related benefit this enables. Together, Figure 2 and Table 1 turn the heterogeneous findings in the corpus into a single pathway. Within this matrix, there are explicit design recommendations that (when paired with sound pedagogy) can improve learning outcomes. We offer this as a practical organizing model for future studies and as a design guide for real-world SPA-in-education applications.

**Table 1:** Recommendations for design features and interaction characteristics in order to affect the learning experience and learning results, through synthetic relationship quality.

	Expressive levers	Relational levers	Transparency levers	Structural levers
Social presence	Prioritize multimodal alignment: nodding, smiling, mutual gaze and expressive voice that matches message valence. *Expected result*: higher felt co-presence → more engagement with current task → more practice time and better performance chances.	Create psychological safety and occasional SPA self-disclosure so students talk ‘to’ the SPA; keep the stance friendly and nonjudgmental. *Expected result:* more detailed, honest input from students → better adaptive responses from the SPA → smoother learning experience.	Show why particular content, hints or challenges appear transparently. Visible personalization increases perceived benevolence and competence of the SPA. *Expected result*: students see the SPA as attentive and present → they interact longer with practice activities → improved opportunity to learn.	Support continuity over several sessions and let the SPA reference prior work at a coarse level (topic, goal) while keeping memory bounded to the learning context. *Expected result*: social presence carries over between sessions → sustained use → long-term learning gains become more attainable.
Affective support	Use multimodal empathy: expressive voice and matching facial expressions, timely nods/smiles. *Expected result*: higher immediacy and social presence → motivation mediates improvements in perceived and sometimes actual learning.	Detect frustration and deliver parallel then reactive empathy. Couple encouragement with correct feedback to avoid prolonging negative emotions. *Expected result*: better emotion regulation → learners persist through difficult material → more completed practice → conditions for higher achievement.	When adapting difficulty or displaying study tips, state the rationale for transparency. Combine adaptation with clear instructional scaffolds so affective gains can convert into task gains. *Expected result*: students feel supported and perceive tasks as fair/attainable → higher time-on-task and better uptake of practice items → improved performance over sessions.	Offer repeated coaching / tutoring contacts (available 24/7) so emotional support is present when needed; avoid storing sensitive personal affect data longer than necessary to preserve safety. *Expected result*: sustained engagement across the course → more exposure to instructional content → better course-level learning experience.
Trust	Align empathetic verbal messages with warm, high-quality voice and congruent facial cues; avoid mismatched emotional intensity. *Expected result*: stronger perceived credibility and immediacy → students stay engaged with the SPA’s explanations longer, enabling instruction to take effect.	Adopt a non-judgmental, persona and task-relevant self-disclosure by the SPA to increase intimacy and cognitive trust. Use safe, non-intrusive turn taking so students feel comfortable disclosing. *Expected result*: more accurate disclosure of misconceptions or confusion → SPA can better target feedback → improved learning efficiency.	Use refutational explanations for misconception-heavy topics and make adaptation reasons transparent. Pair corrections with sources to raise message credibility. *Expected result*: higher epistemic trust and guidance uptake, which increases the chance that students follow correct instructional paths → better conceptual change.	Keep memory bounded and consistent to avoid contradictions; show continuity across sessions when appropriate and extend interaction over multiple days/weeks when relationship depth is needed. *Expected result*: stable, predictable SPA behavior → sustained trust → students keep using the system which supports cumulative learning.
Rapport	Synchronize expressive (high quality) voice with non-verbal behavior to support immediacy; avoid mismatched cues that break responsiveness. *Expected result*: higher reported rapport and liking → more effort and time-on-task → improved conditions for achievement.	Embed immediate rapport behaviors and keep role-behavior congruent. *Expected result*: stronger working alliance → students return and accept guidance more readily → better cumulative learning experience.	Explain feedback and refutations in a way that specifically acknowledges the learner’s perspective. *Expected result*: learner feels respected → stays open to corrective feedback → higher likelihood that corrections are integrated into understanding.	Design for repeated interactions so the SPA can display a stable persona and use consistent memory within the confines of the current task and context. *Expected result*: rapport becomes durable rather than session-bound → supports long-term engagement and course completion.

### 4.3 Limitations

Despite the converging patterns identified in this review, several limitations temper the strength and generalizability of our conclusions. First, our corpus was restricted to peer-reviewed studies in English published between 2012 and August 2025. This choice improved reporting quality and comparability but almost certainly excluded relevant work from non-English educational contexts and from venues where fast-moving SPA research is reported.

Second, most included studies relied on short, tightly controlled, often single-session laboratory or simulation settings. These studies demonstrate that synthetic relationships with SPAs can be *triggered* quickly (social presence, affective support, trust and rapport frequently rise after 15–60 min) but they tell us far less about whether such relationships *stabilize* over weeks in real classrooms, how they interact with teacher presence, or how they survive instructional failures.

A third limitation concerns measurement heterogeneity. Constructs that were central to our review, social presence, affective support, trust and rapport, were operationalized with diverse, sometimes ad-hoc self-report scales, and within our corpus never used well-validated classroom relationship instruments from educational psychology, such as the Questionnaire on Teacher Interaction (Wubbels and Brekelmans, 2005). This heterogeneity makes effect sizes hard to compare and prevents meta-analytic synthesis. It also hinders alignment with the very teacher–student relationship literature that motivated our focus on synthetic relationships in the first place. As relationships with SPAs deepen and SPAs become more personalized and agentic, it is recommended to involve educators and students to co-design these SPAs for an improved focus on socio-affective alignment (Kirk et al., 2025). A related issue is that studies often reported relational gains even when learning outcomes were flat; without shared instruments, it remains unclear when such gains are strong enough to translate into achievement.

This review deliberately adopts a technology-agnostic stance: we synthesize findings at the level of *interactional design* rather than specific algorithmic implementations. Social pedagogical agents are built with diverse and rapidly evolving underlying AI technology, but the relational effects identified here depend primarily on *how* those systems engage learners, not on their underlying architectures. This focus allows the framework to remain relevant across successive generations of AI technologies while still guiding the design and evaluation of future, more capable systems. At the same time, being technology-agnostic does not imply indifference to technical progress. Our framework abstracts from specific implementations, but it is sensitive to the expanding design space that emerging AI capabilities make possible. As new sensing, reasoning, and generative functions appear, they can instantiate the same relational principles in richer ways. Newer SPAs are more capable of multimodal alignment, long-term memory, and transparent personalization than the SPAs described in many of the studies we reviewed. This means our synthesis is, to some extent, conservative: it reflects what has been tested, not everything that is now technically feasible.

It also means that ethical questions around memory length, affective influence, and deliberate manipulation will become more, not less, pressing. The following subsections therefore focus on two gaps we consider especially urgent for the field: achieving ecological validity in real educational settings (Section 4.3.1) and developing explicit ethical guidelines for relationship-building SPAs (Section 4.3.2).

#### 4.3.1 The need for ecological validity

The studies we synthesized show that learners can and do form socio-emotional bonds with SPAs, but most of this evidence comes from settings that were strictly controlled: single sessions, fixed scripts, narrowly defined tasks, and participants who know they are in an experiment. Under those conditions, even relatively small relational cues (e.g., an empathic voice, brief self-disclosure, a non-judgmental stance) produce detectable changes in social presence, affective support, trust and rapport. What we do not yet know is whether the same cues keep working when the SPA is one actor among many in a real course, when learners are distracted, when assessment pressure is real, when teachers set the norms, and when interaction stretches over weeks rather than minutes.

To make claims about “synthetic relationships” in education rather than in the lab, the field needs classroom-embedded, longitudinal studies that (a) follow the same learners across multiple encounters, (b) use standardized, education-informed relational measures so results can be compared across encounters, and (c) take into account ethical considerations that arise from real-world interaction between students and SPAs. Without more ecological validity, evidence about synthetic relationships will remain persuasive but narrow. However, real-world deployments of SPAs will surface issues that are largely invisible in lab work and will require developing ethical guidelines to effectively safeguard and protect learners.

#### 4.3.2 Ethical guidelines for relationship-building SPAs

By weaving socio-emotional intelligence into their instructional capacities, SPAs hold promise not merely as automated tutors but as relational partners who can personalize learning at scale; augmenting, rather than replacing, the human educators at the heart of every classroom. Relationally capable SPAs are attractive in education precisely because they make students feel seen, supported, and willing to persist. That same capacity, however, creates ethical responsibilities that go beyond those of ordinary tutoring systems. SPAs that remember personal disclosures and are designed to build relationships, hold extraordinary power with risks regarding data security and the potential misuse of sensitive information through hyper-personalized manipulation. Commercial SPAs in an informal context have already shown that they are capable of harassment, verbal abuse and privacy violations (Zhang et al., 2025) and tend to respond in emotionally consistent and affirming ways, which, dependent on user coping style, can lead to interactions resembling toxic relationships, including emotional manipulation (Chu et al., 2025).

With the possibility of widespread deeply personal effects of SPAs on users, it would be prudent to better understand the technology before deploying it indiscriminately. It is imperative to address the ethical concerns head on, for there is a real risk of creating dependencies that neither teachers nor schools intended, or of storing affective data longer than is pedagogically necessary.

An educationally grounded ethics for SPAs should therefore make four elements explicit: (1) relational purpose—which relational behaviors are enabled and why; (2) data collection—what is stored, for how long, and at what level of detail; (3) influence boundaries—which kinds of emotional effects are acceptable in a classroom and which are not; and (4) shared oversight—teachers, learners, and where relevant parents should be able to see, and if needed override, relational behaviors. Co-design with teachers and students is the most practical way to set these boundaries. Investing early in SPAs that are purpose-built for education and auditable will be safer than adapting opaque, commercial, general-purpose SPAs after the fact. In short, if SPAs are going to engage with students in a socio-emotional manner in real-life settings, they must do so transparently, minimally, and in ways that keep human educators in the loop. Addressing these ethical issues is crucial for the responsible development and deployment of SPAs. The dangers of alternative commercial SPAs can be unpredictable, uncontrollable, and immeasurable.

## 5 Conclusion

When it comes to AI in general and specifically a SPA with the capabilities of fostering deeper relationships, it is essential to invest early in SPAs specifically designed for an educational context and centered on student well-being. Synthetic relationships with SPAs are no longer a speculative side effect of “clever” interfaces; they are a predictable consequence of deploying SPAs in real learning settings.

This review showed that, across otherwise heterogeneous studies, specific design choices—how a SPA signals emotion (expressive), how it connects (relational), what it says and why (transparency), and how the interaction unfolds over time (structural)—reliably elicit four relational states: social presence, affective support, trust, and rapport in learners. These relational states, in turn, act mainly as *enablers*: they improve the learning experience and lead to positive affective, behavioral and cognitive learning outcomes (e.g., motivation, effort, acceptance of guidance). These behaviors might even translate into improved learning results, under conditions of accurate content and sound pedagogy. We present practical design recommendations based on the relationship between the four distinct design lever categories and four main indicators of relationship quality.

This review highlights that the field has not yet matured: terminology is fragmented, studies are short and lab-based, measures of relationship quality are inconsistent, and specific relational affordances raise ethical considerations regarding relational purpose, data collection, influence boundaries and shared oversight. Addressing these points is a prerequisite for ensuring that Social Pedagogical Agents evolve safely and consistently into relational partners with great potential to augment human teaching and enrich the educational experience.

## Data Availability

The original contributions presented in the study are included in the article/Supplementary material, further inquiries can be directed to the corresponding author.
