# Supplementary material for: Synthetic relationships with Social Pedagogical Agents in education: a scoping literature review
Source: Front Artif Intell. 2026 Mar 2;9:1625438. doi: 10.3389/frai.2026.1625438 (PMC12989599; doi:10.3389/frai.2026.1625438)
Supplement: Supplementary file 1 [file Data_Sheet_1.pdf]

Appendix 1: studies used for analysis

Chiou, E. K., Schroeder, N. L., & Craig, S. D. (2020). How we trust, perceive, and learn from virtual humans: The influence of voice quality. *Computers & Education, 146*, 103756. https://doi.org/10.1016/j.compedu.2019.103756

Cingillioglu, I., Gal, U., & Prokhorov, A. (2024). Running a double-blind true social experiment with a goal oriented adaptive AI-based conversational agent in educational research. *International Journal of Educational Research*, *124*. Scopus. https://doi.org/10.1016/j.ijer.2024.102323

Coleman, S., Lynch, C., Worlikar, H., Kelly, E., Loveys, K., Simpkin, A., Walsh, J., Broadbent, E., Finucane, F., & O'Keeffe, D. (2025). "Digital Clinicians" Performing Obesity Medication Self-Injection Education: Feasibility Randomized Controlled Trial. *JMIR DIABETES*, *10*. https://doi.org/10.2196/63503

Ding, L., Li, T., Jiang, S., & Gapud, A. (2023). Students' perceptions of using ChatGPT in a physics class as a virtual tutor. *INTERNATIONAL JOURNAL OF EDUCATIONAL TECHNOLOGY IN HIGHER EDUCATION*, *20*(1). https://doi.org/10.1186/s41239-023-00434-1

Eiris, R., Wen, J., & Gheisari, M. (2021). Influence of Virtual Human Appearance Fidelity within Building Science Storytelling Educational Applications. *Journal of Architectural Engineering*, *27*(4). Scopus. https://doi.org/10.1061/(ASCE)AE.1943-5568.0000510

Fountoukidou, S., Matzat, U., Ham, J., & Midden, C. (2022). The effect of an artificial agent's vocal expressiveness on immediacy and learning. *Journal of Computer Assisted Learning, 38*(2), 500–512. https://doi.org/10.1111/jcal.12632

Guetterman, T. C., Sakakibara, R., Baireddy, S., Kron, F. W., Scerbo, M. W., Cleary, J. F., & Fetters, M. D. (2019). Medical students' experiences and outcomes using a virtual human simulation to improve communication skills: Mixed methods study. *Journal of Medical Internet Research*, *21*(11). Scopus. https://doi.org/10.2196/15459

Hong, J., Lin, C., & Juh, C. (2024). Using a Chatbot to learn English via Charades: The correlates between social presence, hedonic value, perceived value, and learning outcome. *INTERACTIVE LEARNING ENVIRONMENTS*, *32*(10), 6590–6606. https://doi.org/10.1080/10494820.2023.2273485

Hopman, K., Richards, D., & Norberg, M. M. (2023). A Digital Coach to Promote Emotion Regulation Skills. *Multimodal Technologies and Interaction*, *7*(6). Scopus. https://doi.org/10.3390/mti7060057

Huang, H., Chen, Y., & Rau, P. (2022). Exploring acceptance of intelligent tutoring system with pedagogical agent among high school students. *Universal Access in the Information Society, 21*(2), 381–392. https://doi.org/10.1007/s10209-021-00835-x

Huang, W., Jia, C., Hew, K., & Guo, J. (2024). Using chatbots to support EFL listening decoding skills in a fully online environment. *Language Learning & Technology, 28*(2), 62–90. https://doi.org/10.64152/10125/73572

Ivanović, M., Mitrović, D., Budimac, Z., Jerinic, L., & Bâdicâ, C. (2015). HAPA: Harvester and pedagogical agents in e-learning environments. *International Journal of Computers, Communications and Control*, *10*(2), 200–210. Scopus. https://doi.org/10.15837/ijccc.2015.2.1753

Kim, J., Lee, K., Kim, W., Jeong, N., Kim, J., & Song, H. (2025). Empathetic Pedagogical Agent: Mitigating Harmful Effects of Negative Feedback Through Self-Disclosure. *International Journal of Human–Computer Interaction*, *41*(15), 9366–9383. https://doi.org/10.1080/10447318.2024.2425881

Krämer, N., Karacora, B., Lucas, G., Dehghani, M., Rüther, G., & Gratch, J. (2016). Closing the gender gap in STEM with friendly male instructors? On the effects of rapport behavior and gender of a virtual agent in an instructional interaction. *COMPUTERS & EDUCATION*, *99*, 1–13. https://doi.org/10.1016/j.compedu.2016.04.002

Laverde, N., Grévisse, C., Jaramillo, S., & Manrique, R. (2025). Integrating large language model-based agents into a virtual patient chatbot for clinical anamnesis training. *Computational and Structural Biotechnology Journal*, *27*, 2481–2491. Scopus. https://doi.org/10.1016/j.csbj.2025.05.025

Liao, M., Luo, X., Yang, H., & Zhu, K. (2024). The interactive effects of pedagogical agent role and voice emotion design on children's learning. *CURRENT PSYCHOLOGY*, *43*(36), 29170–29188. https://doi.org/10.1007/s12144-024-06559-4

Moridis, C. N., & Economides, A. A. (2012). Affective Learning: Empathetic Agents with Emotional Facial and Tone of Voice Expressions. *IEEE Transactions on Affective Computing*, *3*(3), 260–272. https://doi.org/10.1109/T-AFFC.2012.6

Nebel, S., Beege, M., Schneider, S., & Rey, G. D. (2020). Competitive Agents and Adaptive Difficulty Within Educational Video Games. *Frontiers in Education*, *5*. https://doi.org/10.3389/feduc.2020.00129

Nelekar, S., Abdulrahman, A., Gupta, M., & Richards, D. (2022). Effectiveness of embodied conversational agents for managing academic stress at an Indian University (ARU) during COVID-19. *British Journal of Educational Technology*, *53*(3), 491–511. https://doi.org/10.1111/bjet.13174

Oker, A., Pecune, F., & Declercq, C. (2020). Virtual tutor and pupil interaction: A study of empathic feedback as extrinsic motivation for learning. *Education And Information Technologies*, *25*(5), 3643–3658. https://doi.org/10.1007/s10639-020-10123-5

Pataki, C., Pato, M. T., Sugar, J., Rizzo, A. S., Parsons, T. D., George, C. S., & Kenny, P. (2012). Virtual patients as novel teaching tools in psychiatry. *Academic Psychiatry*, *36*(5), 398–400. https://doi.org/10.1176/appi.ap.10080118

Poeschl, S. (2017). Virtual reality training for public speaking-A QUEST-VR framework validation. *Frontiers in ICT*, *4*(JUN). https://doi.org/10.3389/fict.2017.00013

Ryan, L., Coleman, S., Zimmermann, T., Coyne, R., Broadbent, E., Browne, A., O'Donoghue, G., Quigley, F., Worlikar, H., Connolly, C., Crotty, M., Birney, S., Conlan, O., Walsh, J., & O'Keeffe, D. (2025). A Pilot Feasibility Study Exploring the Preliminary Effectiveness of an AI-Driven Virtual Human Intervention for General Practitioner Obesity Education and Communication-Skills Training. *OBESITY SCIENCE & PRACTICE*, *11*(4). https://doi.org/10.1002/osp4.70083

Sanjeewa, R., Iyer, R., Apputhurai, P., Wickramasinghe, N., & Meyer, D. (2025). Perception of empathy in mental health care through voice-based conversational agent prototypes: Experimental study. *JMIR Formative Research, 9*, e69329. https://doi.org/10.2196/69329

Savin-Baden, M., Tombs, G., Burden, D., & Wood, C. (2013). "It's almost like talking to a person": Student disclosure to pedagogical agents in sensitive settings. *International Journal of Mobile and Blended Learning*, *5*(2), 78–93. https://doi.org/10.4018/jmbl.2013040105

Schmidt, S., Köysürenbars, I., & Steinicke, F. (2024). Frankenstein's Monster in the Metaverse: User Interaction With Customized Virtual Agents. *IEEE Transactions on Visualization and Computer Graphics*, *30*(11), 7162–7171. https://doi.org/10.1109/TVCG.2024.3456205

Schroeder, N. L., Chiou, E. K., Siegle, R. F., & Craig, S. D. (2023). Trusting and learning from virtual humans that correct common misconceptions. *Journal of Educational Computing Research, 61*(4), 790–816. https://doi.org/10.1177/07356331221139859

Sikström, P., Valentini, C., Sivunen, A., & Kärkkäinen, T. (2024). Pedagogical agents communicating and scaffolding students' learning: High school teachers' and students' perspectives. *Computers & Education, 222*, 105140. https://doi.org/10.1016/j.compedu.2024.105140

Wang, Y., Gong, S., Cao, Y., & Liu, Y. (2025). Parallel empathy or reactive empathy? The role of emotional support provided by affective pedagogical agent in online learning. *The Internet And Higher Education*, *67*, 101035. https://doi.org/10.1016/j.iheduc.2025.101035
